# Scale-free correlations and potential criticality in weakly ordered populations of brain cancer cells

**DOI:** 10.1126/sciadv.adf7170

**Published:** 2023-06-28

**Authors:** Kevin B. Wood, Andrea Comba, Sebastien Motsch, Tomás S. Grigera, Pedro R. Lowenstein

**Affiliations:** ^1^ Department of Biophysics, University of Michigan, Ann Arbor, MI, USA.; ^2^ Department of Physics, University of Michigan, Ann Arbor, MI, USA.; ^3^ Department of Neurosurgery, University of Michigan, Ann Arbor, MI, USA.; ^4^ Rogel Cancer Center, University of Michigan Medical School, Ann Arbor, MI, USA.; ^5^ School of Mathematical and Statistical Sciences, Arizona State University, Tempe, AZ, USA.; ^6^ Instituto de Física de Líquidos y Sistemas Biológicos (IFLySiB), Buenos Aires, Argentina.; ^7^ Universidad Nacional de La Plata, La Plata, Buenos Aires, Argentina.; ^8^ CONICET, Godoy Cruz, Buenos Aires, Argentina.; ^9^ Departamento de Física, Universidad Nacional de La Plata, La Plata, Buenos Aires, Argentina.; ^10^ Istituto dei Sistemi Complessi, Consiglio Nazionale delle Ricerche, Rome, Italy.; ^11^ Department of Cell and Developmental Biology, University of Michigan, Ann Arbor, MI, USA.; ^12^ Department of Biomedical Engineering, University of Michigan, Ann Arbor, MI, USA.

## Abstract

Collective behavior spans several orders of magnitude of biological organization, from cell colonies to flocks of birds. We used time-resolved tracking of individual glioblastoma cells to investigate collective motion in an ex vivo model of glioblastoma. At the population level, glioblastoma cells display weakly polarized motion in the (directional) velocities of single cells. Unexpectedly, fluctuations in velocities are correlated over distances many times the size of a cell. Correlation lengths scale linearly with the maximum end-to-end length of the population, indicating that they are scale-free and lack a characteristic decay scale other than the size of the system. Last, a data-driven maximum entropy model captures statistical features of the experimental data with only two free parameters: the effective length scale (*n_c_*) and strength (*J*) of local pairwise interactions between tumor cells. These results show that glioblastoma assemblies exhibit scale-free correlations in the absence of polarization, suggesting that they may be poised near a critical point.

## Teaser

Glioma cells exhibit scale-free correlations in the absence of order, compatible with their being poised at criticality.

## INTRODUCTION

Glioblastoma is the most aggressive brain cancer and remains the cancer with worst prognosis and shortest life expectancy. The standard of care treatment consists of resective surgery, radiotherapy, and chemotherapy. Long-term survival, nevertheless, has remained stagnant over the past 30 years despite major research efforts and multiple clinical trials (*1*, *2*). Even when the glioblastoma tumor is resected, it always recurs, usually within a 1- to 2-cm margin of the original resection cavity. The tumor only rarely metastasizes to distant organs but invades the surrounding normal brain, destroying normal brain areas and disrupting brain function (*3*). Glioblastoma is the sole cancer that kills by direct invasion of surrounding normal brain tissue rather than through metastasis of distant organs. Understanding the mechanisms of glioblastoma growth and invasion are thus paramount to any future successful treatment of this disease.

Collective motion in brain tumors has been studied in vitro and in vivo. However, our understanding of the mesoscale organization of brain tumor collective motion has not been studied in sufficient detail. In particular, collective motility requires close behavioral coordination between individual cells (*4*–*7*). Such coordination could be implemented either by direct cell-to-cell contact, by close but indirect contact, or through long-range information exchange between cells (*8*, *9*). The potential existence of such long-range communication might allow tumor cells to respond quickly to a number of insults and thus provide robustness to tumor growth and progression. However, it is not clear whether such long-range correlations exist in brain tumors, and if so, whether these large-scale patterns actually arise from local interactions between nearby cells.

Collective motion arises in a large range of biological systems, from flocks of birds (*10*–*14*) to schools of fish (*15*, *16*), from single cells (*17*, *18*) and insects (*19*–*23*) to populations of mammals (*24*, *25*), and has been intensely studied for decades in both natural systems (*26*–*28*) and robotics (*29*). Centralized, top-down mechanisms, for example, the presence of one or more “leaders” that dictate the behavior of the others (*30*, *31*), could drive large-scale order, leading to synchronized motion between different cells or organisms. On the other hand, populations may exhibit emergent self-organization driven by local interactions between cells (*26*–*28*), leading to signatures of collective behavior even when motion appears only weakly polarized. Despite the apparent lack of order, self-organized systems can respond coherently to external perturbations, for example, the presence of a predator in animal flocks, because local interactions facilitate an exquisite sensitivity to the changing environment, effectively propagating information from one region of the flock to another in a manner unseen in top-down organizational schemes (*10*, *32*, *33*).

One clear signature of collective behavior is the presence of scale-free correlations, which have been observed in a number of biological systems (*32*, *34*), including many exhibiting collective motion (*10*, *13*). Correlations are considered scale-free when they lack a characteristic decay scale other than the size of the system. For example, in starlings, scale-free correlations in directional velocity occur in highly polarized flocks, where the distribution of velocity angles is extremely narrow (*10*, *12*). Similar scale-free correlations arise in physical systems with a continuous symmetry, in this case, the rotational invariance of the velocity angle, where low-energy fluctuations give rise to Goldstone modes such as long-wavelength spin waves (*35*). By contrast, long-range correlations can also arise in the absence of order, for example, swarming midges exhibit scale-free correlations despite showing only weak levels of directional polarization (*21*). In physics, similar phenomena can occur when system parameters are tuned to a so-called critical point, where fluctuations become correlated across length scales and the system becomes exquisitely sensitive to small perturbations (*35*). Out of equilibrium, scale-free correlations can also appear as a consequence of a conservation law (*36*) or through a feedback mechanism (*34*, *37*, *38*). Recent work has argued for the existence of criticality in a wide range of biological systems (*32*, *39*), including synchronization in neural ensembles (*40*, *41*), brain activity (*42*), long-range speed correlations in starling flocks (*13*), the dynamics of biochemical networks (*43*) and protein folding (*44*), and the scale-free correlations in swarming midges (*21*). Independently of their origin, long-range correlations could potentially serve different biological functions. For example, in flocks of birds, collective behavior may have evolved to promote lossless information flow throughout the group as they move through space, while swarms of midges might use collective behavior to stabilize their behavior against environmental perturbations, such as a predator.

In this work, we quantitatively characterize collective motion in an ex vivo experimental model of glioblastoma using time-resolved tracking of individual glioma cells. We found that populations of glioma cells exhibit collective motion characterized by weak polarization in the (directional) velocities of single cells, suggesting, on the surface, that glioma cells may be moving largely independently. However, by examining populations of different sizes, we observed correlated fluctuations on scales many times the size of a single cell, with characteristic (correlation) lengths that increase linearly with the total system size, showing that they are scale-free. Last, we show that statistical features of the populations are well captured with a simple maximum entropy model, which reduces to the classical *XY* model with nonlocal coupling, which describes the shape of the directional velocity distributions and the existence of long-range correlations. Our results indicate that beneath a weakly ordered facade, brain tumor assemblies exhibit scale-free features of collective behavior on scales of millimeters or more.

## RESULTS

We analyzed glioma cell dynamics in a previously developed ex vivo, explant model derived from the orthotopic implantation of genetically engineered NPA–green fluorescent protein–positive (NPA-GFP^+^) cells, in which we can track the location and velocity of fluorescently labeled individual glioma cells for up to 2 or 3 days (Materials and Methods). Briefly, this experimental model enables de novo the induction of glioma tumors through the injection of different plasmids encoding (i) driver genes found in human gliomas and (ii) genes encoding for luminescent and fluorescent markers in postnatal day 1 (P01) wild-type C57BL/6 mice. When animals became symptomatic, tumors were removed, and neurosphere cell cultures were established as described earlier (*45*–*47*). Cells from these neurosphere cultures can then be implanted into adult C57BL/6 mice to reliably generate tumors (see Materials and Methods for details on the establishment of NPA tumors). Specifically, we implanted NPA cells into the brains of adult C57BL/6 mice (*4*, *45*, *46*). Animals were euthanized at day 19 after tumor implantation to generate explant brain tumor slices for time-lapse imaging on a laser scanning confocal microscope equipped with a tissue culture incubation chamber. We studied a collection of 12 different glioblastoma populations drawn from eight different explants: four from the inner tumor and four from the outer tumor region bordering the normal surrounding brain. We subdivided each explant into regions based on a previously developed classification scheme for glioblastoma cells (*4*). While this scheme was originally developed to classify subpopulations of cells based on histological and statistical properties of the cell orientations, in the present context, the subdivision scheme can be viewed as a box-like approximation for estimating finite-size scaling in the experimental system.

### Weakly ordered directional motion in glioblastoma populations

**Fig. 1.**
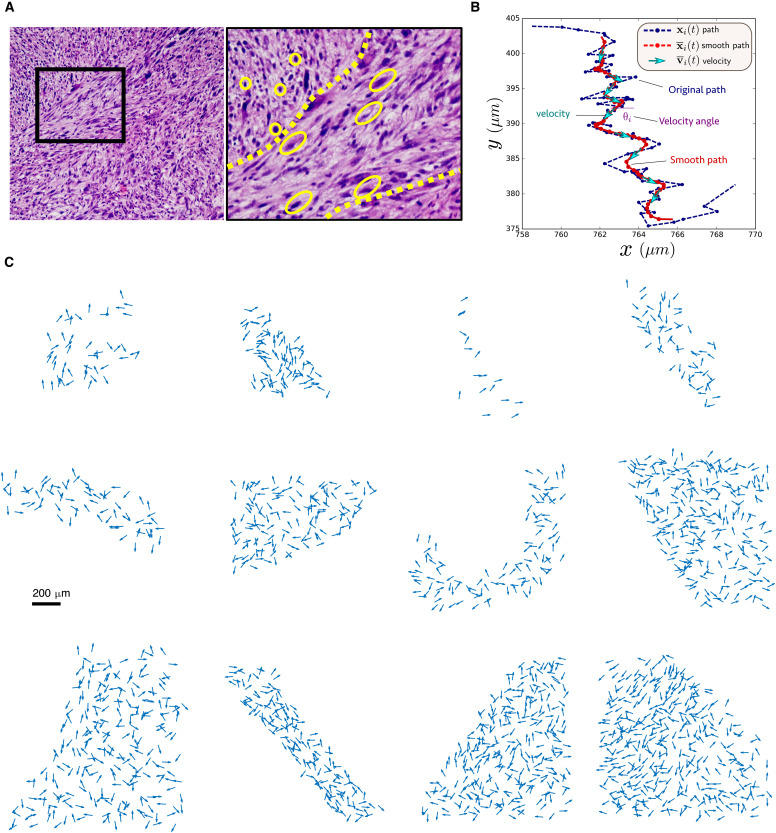
Tracking cell velocities in glioblastoma. (**A**) Representative image of a 5-μm hematoxylin and eosin–stained sections from an NPA mouse glioma tumor. The left image has been taken at lower power, and the black box is shown at higher power on the right. Oblong yellow outlines indicate elongated cells within an oncostream, which refers to a bundle of spindle-like cells that move throughout brain tumors (*4*, *7*). Round yellow outlines indicate round cells located outside the oncostream. (**B**) Example of path obtained with ImageJ (blue dashed line) and the filtered path (red) obtained after filtering. The smooth path allows us to estimate the velocity of the cell at each time step. (**C**) Snapshot of unit velocity vectors in 12 different populations of different sizes.

**Fig. 2.**
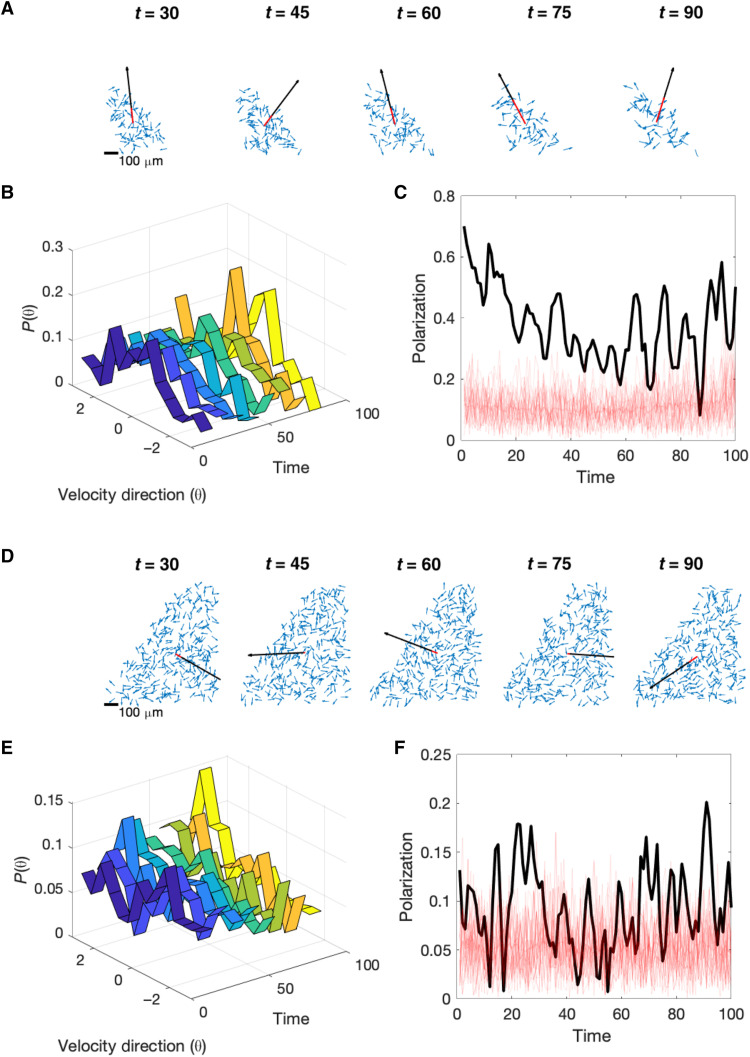
Cell velocities exhibit weak directional order. (**A**) Snapshot of unit velocity vectors (blue) and average velocity vector (red) at different time points. The black arrow is a reference vector indicating full alignment (polarization = 1); length of red vector relative to black vector indicates polarization. (**B**) Distribution of velocity directions over time (dark blue, early times; yellow, late times). (**C**) Polarization over time for glioblastoma populations (black) and for 25 simulated datasets (size-matched to actual data) but with velocity directions drawn from a uniform distribution (light red; mean over all datasets in dark red). Polarization is defined by *S* = ∣〈**s**〉∣ ≡ ∣(1/*N*)∑*_i_* ‍ **s***_i_*∣, where ∣**x**∣ is the length of vector **x** and angle brackets indicate an average over all cells. (**D** to **F**) Identical to (A to C) but for a second dataset. See the Supplementary Materials for all datasets.

To quantify the population-level motion in the tumor, we estimated the position and velocity of each cell using semiautomated image analysis (Fig. 1; Materials and Methods). At each time point, the population is described by a set of normalized (unit) velocity vectors {**s**_***i***_(*t*)} and a corresponding set of position vectors {**x**_***i***_(*t*)}, one for each cell *i* = 1,2…*N*. To quantify the degree of global ordering in the population, we calculated the polarization *S*(*t*), which is defined as the magnitude of the population’s mean velocity; *S* = 1 when all cells move in the same direction, while *S* = 0 if the velocities are uniformly distributed in all directions. We found that glioblastoma populations are weakly polarized, often exhibiting levels of polarization comparable to, or only slightly exceeding, those in size-matched populations with velocities randomly drawn from a uniform distribution (compare black and red curves; Fig. 2 and fig. S1). This weak polarization corresponds to broad but typically unimodal distributions of directional velocity (Fig. 2 and fig. S2). In contrast to starling flocks, which are highly polarized (*10*, *12*), glioblastoma populations show minimal levels of polarization and therefore appear disordered at the population level. Such weak polarization could be evidence that cells move largely independently, with little functional coupling between cells; on the other hand, weak polarization, alone, is insufficient to rule out collective behavior. Populations weakly ordered on the global scale have been shown to exhibit features of long-range collective behavior in a number of biological contexts, including swarming behavior of midges (*48*) and synchronous firing activity in neural populations (*49*, *50*). In these cases, fluctuations can be correlated over long spatial distances, even when ordering (i.e., polarization) is weak.

### Glioblastoma populations exhibit scale-free correlations in velocity fluctuations

To determine whether glioma populations exhibit correlated spatial fluctuations, we calculated correlations between directional velocity for cells separated by a given distance (*r*; in micrometers) for populations with different (time-averaged) spatial sizes (*L*), where the size of the system is defined as the largest linear dimension of the population, that is, the maximum separation between any pair of cells. Correlation functions are defined by *C*(*r*) = 〈δs*_i_* · δs*_j_*〉*_r_*, where δs*_i_* ≡ s*_i_* − 〈s〉 is the velocity of cell *i* in the moving reference frame where the population average velocity (〈s〉) has been subtracted out. Angle brackets 〈〉*_r_* indicate an average taken over all cells separated by distance *r*.

**Fig. 3.**
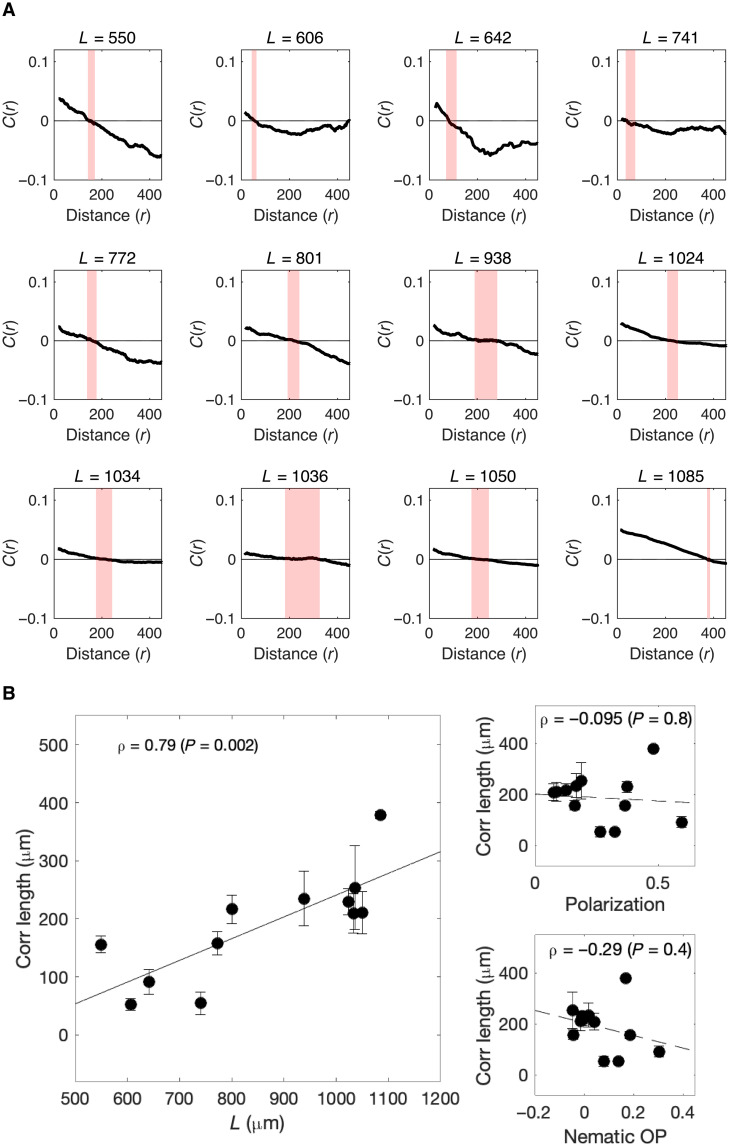
Velocity fluctuations are correlated over a length scale that depends on system size. (**A**) Correlations between directional velocity for cells separated by a given distance (*r*; in micrometers) for populations with different (average) spatial sizes (*L*). Correlation functions are defined by *C*(*r*) = 〈δ*s_i_* · δ*s_j_*〉*_r_*, where δ*s_i_* ≡ *s_i_* − 〈*s*〉 is the velocity of cell *i* in the moving reference frame where the population average velocity (〈*s*〉) has been subtracted out. Angle brackets 〈〉*_r_* indicate an average taken over all cells separated by distance *r*. Black curves: time-averaged correlations in a given population; red dashed line: estimated correlation length *r*_0_, which corresponds to the crossover point *C*(*r*_0_) = 0. (**B**) Main panel: Correlation length (*r*_0_) for subpopulations of different sizes (black circles; error bars represent uncertainty in estimate of crossover point). The solid line is best-fit line (for visualization). Right: Correlation length versus polarization (top) and nematic (bottom) order parameters for the populations in (A). The dashed line is best-fit line. Nematic order parameter is defined as Nematic OP=2⟨cos^2^θ⟩ − 1, where θ defines the orientation of the mean spin. Insets show Pearson correlation coefficient (ρ) and corresponding *P* value.

We found that all populations exhibit weak local correlations over tens of micrometers (Fig. 3). To characterize the decay of these correlations over space, we computed *r*_0_ [i.e., the point at which *C*(*r*) first crosses 0] for populations of different sizes. We found that *r*_0_ depends approximately linearly on the spatial size (*L*) of each population. This linear relation is expected to hold when the correlation length is much larger than the size of the system [i.e., when the correlated fluctuations are scale-free (*51*)], and *r*_0_ can be interpreted as a correlation length scale; in contrast, when the correlation length is less than the system size, *r*_0_ grows as log*L*. We note that similar scaling is observed when system size is defined using other metrics (fig. S4) and for different levels of smoothing (fig. S3). By contrast, order parameters, including both polarization and an order parameter for nematic ordering in 2D, are not correlated with the size of the system (Fig. 3).

While the scaling analysis above suggests that correlations in velocity fluctuations are scale-free, it is limited to the relatively small number of sample populations, whose sizes span approximately a factor of two, and correlation lengths (strictly, *r*_0_) estimated from these samples are inherently noisy. To complement this analysis, we performed a “box scaling” analysis (see Materials and Methods), a powerful approach to finite-size scaling built on subsampling data in randomly chosen spatial boxes of different sizes (*52*). To do so, we randomly selected many subpopulations of cells from all datasets using square boxes of a fixed width *W* placed randomly (with uniform distribution) within the field of view. Data from boxes of a given width were then combined and used to estimate correlation functions and crossover points *r*_0_ for these different-sized subsystems. This approach is advantageous because (i) repeated subsampling allows us to estimate *C*(*r*) and *r*_0_ more precisely by dividing data into a large number of smaller samples, (ii) the analysis does not depend on a priori division of datasets into subpopulations based on histological or other characteristics, and (iii) it allows us to explore scaling behavior over a wider range of system sizes. Previous studies have shown that the approach provides an excellent approximation to a full scaling analysis in both classical statistical physics models and real datasets, even when a wide range of system sizes is not available (*52*).

**Fig. 4.**
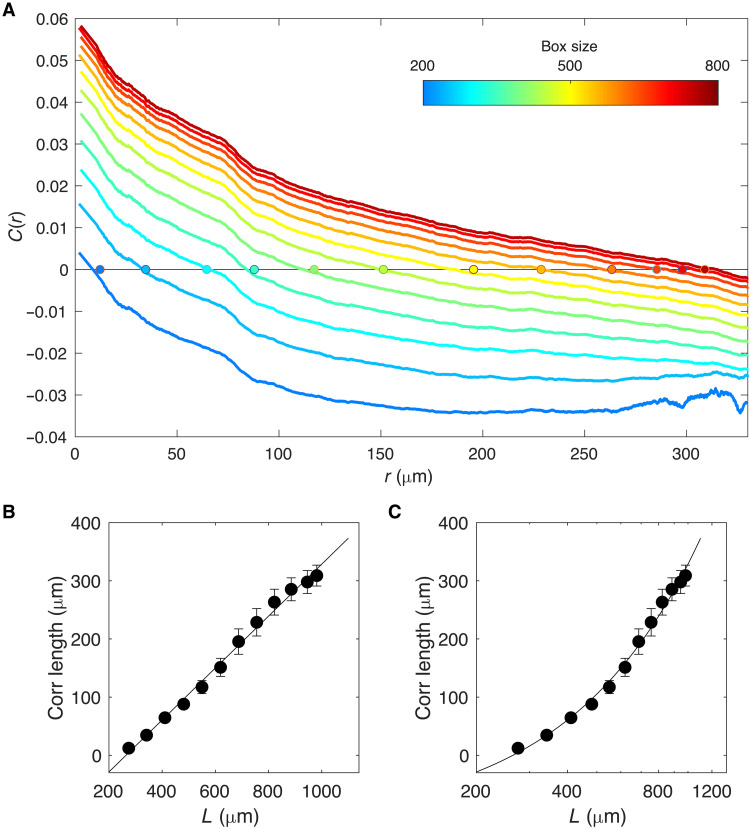
Box scaling indicates correlations in velocity fluctuations are scale-free. (**A**) Correlations between directional velocity for cells separated by a given distance (*r*; in micrometers) for subsampled populations in boxes of different sizes [ranging from 200 μm by 200 μm (blue) to 800 μm by 800 μm (red)]. Filled circles: estimated correlation length *r*_0_, which corresponds to the crossover point *C*(*r*_0_) = 0. (**B**) Correlation length (*r*_0_) for subsampled populations of different sizes (black circles) on linear-linear (B) and log-linear (**C**) scales. Error bars represent uncertainty in estimate of crossover point. Solid line is best-fit line (for visualization).

The box scaling analysis reveals a clear dependence of *C*(*r*) on the box size chosen for sampling (Fig. 4, top), and the characteristic length *r*_0_ depends linearly on the system size (Fig. 4, left). For comparison, we also show the plot on a log-linear scale (Fig. 4, right), which allows us to rule out logarithmic scaling with system size (*r*_0_ ∼ log *L*) that would indicate a correlation length smaller than the system size (*52*). The results are similar if we use alternative measures of system size such as the linear length of the boxes used for scaling (fig. S6). As with the original datasets, the box scaling data exhibits only weak ordering as measured by polarization or nematic order parameters (fig. S6). Together, these findings indicate that glioma populations exhibit collective behavior, specifically, scale-free correlations, in the absence of strong ordering.

### A data-driven maximum entropy model captures statistical features of glioma migration

**Fig. 5.**
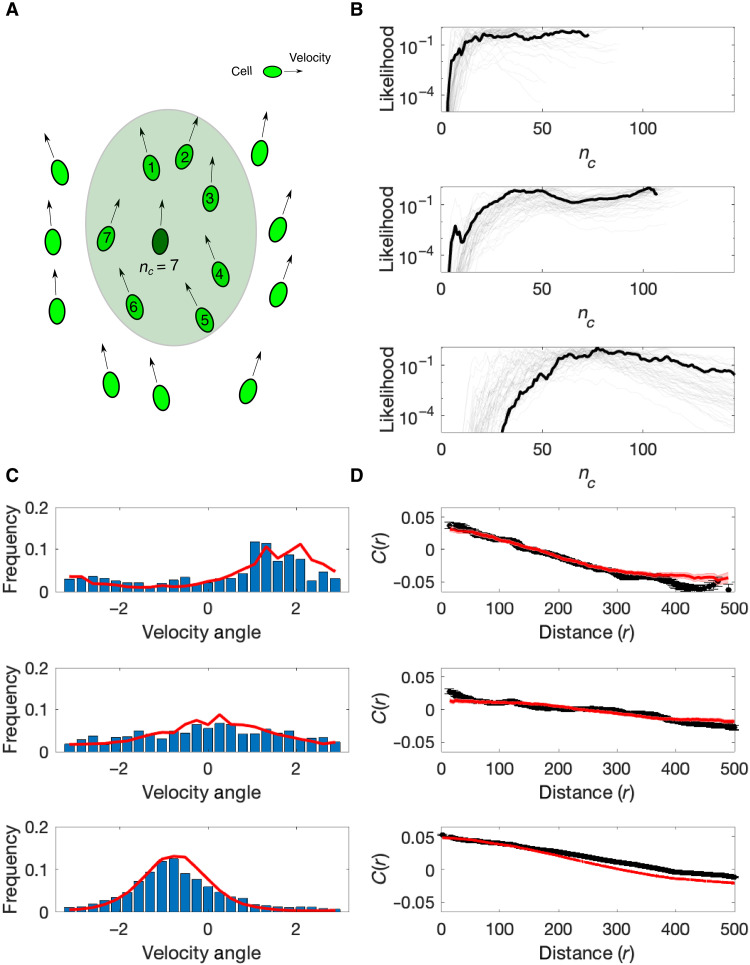
Maximum entropy model captures statistical features of glioblastoma populations. (**A**) Moving cells (ovals, with arrows representing velocity) interact pairwise with the closest *n_c_* neighbors in their local community. Shaded region indicates range of interaction for a single focal cell (dark green) for an illustrative value of *n_c_* = 7. A coupling parameter *J* indicates the strength of interaction between pairs of cells, with larger *J* favoring stronger directional alignment. (**B**) Likelihood (normalized) for the interaction range (*n_c_*) for three different populations; light curves show individual time points, and dark curves are averages over time. (**C**) Histograms of velocity direction (angle) for experiment (blue) and the maximum entropy model (red) for the same three populations as in (B). (**D**) Correlation functions calculated from experimental data (black) and for the maximum entropy model (red) for the same three populations as in (B). See also figs. S7 and S8 for full results on all populations.

To develop a minimal effective model of glioma collective motion, we used the experimental velocity data to parameterize a simple maximum entropy model (Fig. 5A). Maximum entropy methods have been widely applied to model biological phenomena (*32*, *53*), including the collective firing activity of neurons (*49*, *50*); the flocking behavior of birds (*12*, *13*); and correlations in antibody diversity (*12*), drug interactions (*54*), or sequence motifs in biological polymers (*55*). Maximum entropy approaches are closely connected to classical “inverse problems” in statistical physics, which involve estimating (often unobservable) microscopic parameters from a set of macroscopic observables (*56*–*59*). The maximum entropy approach allows one to incorporate a specific set of experimental observables into a model but, in a strict statistical sense, does not introduce additional structure. At its core, a maximum entropy model is consistent with a defined set of measurements but is otherwise as “unbiased” as possible. In the case of tumor cell populations, we would like to determine whether a minimal model that incorporates pairwise interactions of a fixed length scale is sufficient to reproduce the large-scale order observed in experiments. Following (*12*), we developed a maximum entropy model consistent with the local correlation structure measured in experiments, a quantity that we refer to as *C*_int_. *C*_int_ is a single number that describes the average correlation of a cell’s velocity with that of its *n_c_* closest neighbors; if cells tend to be directionally aligned with their neighbors, then *C*_int_ approaches 1, while *C*_int_ is 0 if directional velocities are random.

The maximum entropy model consistent with *C*_int_ is formally identical to a nonlocally coupled version of the *XY* model, which was originally developed in statistical physics to describe systems such as superconductors characterized by a continuous (planar) symmetry (*35*). The model contains two free parameters: the length scale over which cells interact (*n_c_*) and the strength of those interactions (*J*). Our goal is to determine whether such a minimal model, containing only two scalar parameters, can reproduce measured features of the cell ensembles.

The model itself is probabilistic, that is, it specifies the probability of observing a particular set of velocities given any specific choice of the parameters *J* and *n_c_*. In physics parlance, *J* and *n_c_* are “microscopic parameters” that describe physical interactions between the constituent parts of the system (e.g., a parameter analogous to *J* is used to describe the interactions between individual magnetic dipoles in a ferromagnet). Particular choices of these parameters give rise to different types of “macroscopic” behavior, quantities, like polarization, that reflect measurable properties of the system as whole. The mapping from microscopic parameters (*J*, *n_c_*) to macroscopic observables (polarization and correlation functions) is often not straightforward, although statistical physics provides a number of tools for calculating the latter from the former.

We estimated parameters (*J*, *n_c_*) for each population at each time point using a spin-wave approximation to calculate the partition function *Z* (see Materials and Methods). We note that while the spin-wave approximation is strictly valid only in highly polarized systems, we found that the model with these parameter estimates qualitatively captures many features of our data. Different populations are characterized by widely variable (time-averaged) values of *n_c_*, with length scales ranging from tens of cells, in some cases, to hundreds of cells in others, nearly the size of the entire population (Fig. 5B). To compare the model with experimental data, we used Monte Carlo simulations to estimate the distribution of velocity angles (Fig. 5C) and correlation functions (Fig. 5D) for the model with experimentally determined values of *J* and *n_c_*. Despite the simplicity of the model, it captures qualitative features of both the angular distribution and the correlation functions for nearly all populations without additional tuning of free parameters (Fig. 5; see also figs. S7 and S8).

Because the model suggests that cells are coupled over large length scales, with *n_c_* sometimes approaching the size of the population, we also consider a mean field (all-to-all coupled) version of the model. In this case, the model is characterized by a single parameter (*J*), and estimating *J* from experimental data reduces to a classic inverse problem in statistical physics. The advantage of this simpler model is that parameter estimation no longer relies on the spin-wave approximation, and instead, the maximum likelihood estimate of *J* can be calculated analytically in terms of the experimental observable *C*_int_ (Material and Methods). Somewhat unexpectedly, the mean field model captures the angular distribution of velocities nearly as well as the full model (fig. S9). As a numerical control, we confirmed that such agreement does not occur in randomly generated datasets of size-matched populations, confirming that the qualitative agreement between the model and experimental data is unlikely to arise from finite-size statistical effects (fig. S11).

### Effective model of glioma dynamics is poised at a critical point

Glioma cells exhibit scale-free correlations in the absence of polarization. One explanation for this observation is that the system is poised near a critical point. We therefore asked whether the effective maximum entropy model is also poised near a critical point: that is, whether the experimentally derived parameters (*J*, *n_c_*) correspond to a critical point of a model. The absence of criticality in such a model may point to alternative explanations for the experimental observations.

To characterize the phase diagram for the maximum entropy model (i.e., an *XY* model with nonlocal coupling determined by *J* and *n_c_*), we used Monte Carlo simulations to generate representative velocity distributions for different values of *n_c_* and *J*. For each parameter pair, we calculated the polarization order parameter (the mean polarization across trials), the generalized susceptibility χ, which corresponds (up to a proportionality constant) to the variance of the polarization, and the heat capacity, which measures fluctuations in the effective energy. We then estimated the critical surface in the (*J*, *n_c_*) plane to be the curve in parameter space that corresponds to a peak in χ. We also verified that the system exhibits sharply increasing polarization and a peak in the generalized heat capacity at the critical surface. For all simulations, cell positions (and, therefore, cell density) were taken from a representative experimental dataset.

**Fig. 6.**
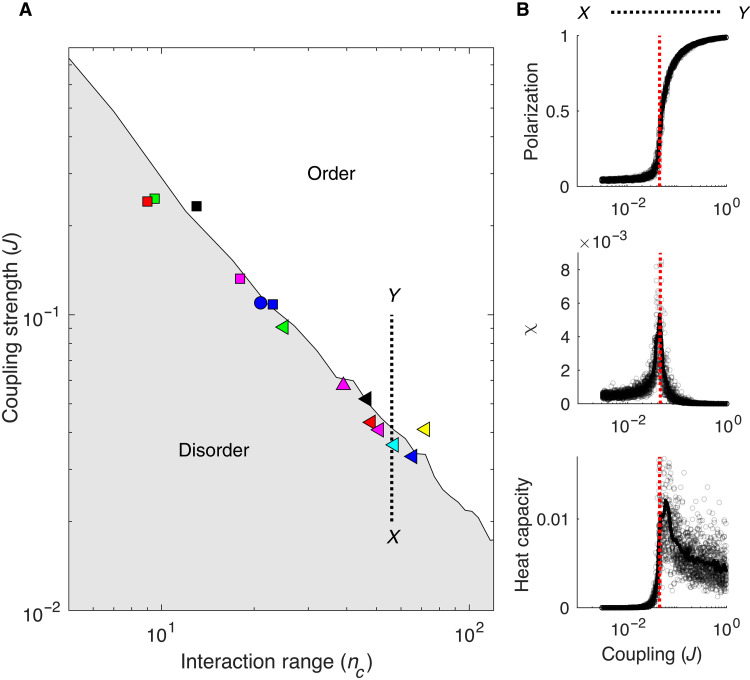
Experimental estimates of cell-cell coupling parameters are poised near critical point of nonlocally coupled *XY* model. (**A**) Phase diagram shows regions of disorder (gray) and order (white) for different values of the coupling strength (*J*) and interaction range (*n_c_*). Phase boundary was estimated from Monte Carlo simulations of the maximum entropy model (Materials and Methods). Markers (squares, triangles, and circles) show estimates of *J* and *n_c_* from different glioblastoma populations. The dashed black line shows an example trajectory through parameter space, in this case, from a point *X* in the disordered region to a point *Y* in the ordered region, that crosses the critical surface by increasing *J* at a fixed value of *n_c_*. (**B**) Polarization (top), generalized susceptibility χ (middle), and generalized heat capacity (bottom) calculated from Monte Carlo simulations of the model as parameters are varied along the dashed trajectory connecting points *X* and *Y* in (A). Gray circles represent Monte Carlo simulations across a range of coupling strengths (*J*) at fixed *n_c_* = 58 (corresponding to the estimated *n_c_* for one representative dataset); solid lines are averages over a total of 100 simulations. The dashed red line indicates estimated value of coupling *J* for one particular dataset [(green triangle in (A)] that lies near the critical surface. Susceptibility (χ) and heat capacity are defined as the variance of polarization and energy, respectively, across independent realizations.

The phase diagram (Fig. 6A) is divided by the critical surface into an ordered region (upper right) and disordered region (lower left, gray). To investigate whether the experimental populations are poised near the critical surface, we plotted the estimated parameter values (*J* and *n_c_*) for each population (triangles, squares, and circles) on the phase diagram. We find that all populations are characterized by parameter pairs that lie close to the critical surface. Modulating the parameters, for example, by simulating a system with a fixed (experimentally determined) value of *n_c_* but *J* values that differ from subcritical to supercritical (as indicated by the dashed line connecting points *X* and *Y*; Fig. 6A), indicates that the parameters estimated from experiments occur near a critical point, where polarization begins to rapidly increase and χ and the heat capacity peak (Fig. 6B).

We stress, however, that criticality in this model alone is insufficient to infer criticality in the glioma populations, both because of technical limitations in distinguishing critical and subcritical systems (see the Supplementary Materials) and, more importantly, because this model may not correspond to the precise physical mechanisms underlying the motion. Nevertheless, the maximum entropy model accurately captures qualitative behavior of the glioma populations and, in doing so, provides a parsimonious effective model for the data. While the overall data suggest that glioma tumors exhibit scale-free correlations in the absence of ordering, which is compatible with criticality, further experiments will be needed to determine brain tumor criticality beyond doubt.

## DISCUSSION

Our data suggest that the brain tumors exhibit features of large scale collective behavior in the absence of strong order. One potential explanation is that glioma populations are poised near a critical point, that is, on the boundary between an ordered and disordered phase. A growing number of studies suggest that biological systems may operate at or near criticality (*13*, *32*, *34*, *40*–*44*, *60*–*62*). However, scale-free correlations can also be found in some systems that are noncritical. For example, scale-free correlations have been observed in strongly ordered animal flocks (*12*, *26*), and similar behavior is expected in equilibrium systems with a continuous symmetry (*63*). This explanation does not apply to glioblastoma cells, however, because they are largely disordered (the order parameter of the subsystems is rather small; Fig. 2).

Out of equilibrium, it is possible to build models that display long-range, scale-free correlations without a tuning parameter, for example, in systems with a conservation law (*36*) or in self-organized criticality (SOC) models (*34*, *37*, *38*), where a feedback mechanism keeps the system at the edge between a state with high but fluctuating local activity and an absorbing inactive state. In the case of glioma cells, we cannot see such SOC-like mechanisms obviously at work, although these or other mechanisms might ultimately underlie the scale-free behavior that we observe.

What is the potential advantage of such “disordered” collective behavior? Past work suggests that these properties confer a number of potential biological advantages, including increased sensitivity to environmental stimuli, a large dynamic range, and improved capacity to store and process information (*39*). In the case of glioma cells, these populations may be able to rapidly access a larger set of behaviors when faced with growth or chemotherapy insults. Strongly ordered motion imposes a rigid structure that limits the sensitivity of the population to new environmental insults, such as attacks from the immune system. On the other hand, cells that move entirely independently and lack the ability to move collectively, might represent cells best situated to invade surrounding normal tissue and could also facilitate immune escape. The ideal tumor state may therefore be one that exhibits features of both order and disorder, where tumors may be poised to modulate their capacity for motility, as dictated by clues of the tumor microenvironment.

It is important to keep in mind several limitations of our study, both technical and conceptual. First, the experiments are performed in an ex vivo model, which allowed us to measure cell velocities and track movement for up to 72 hours. As always, however, one should use caution when extrapolating from laboratory results to in vivo dynamics, although our recent data have demonstrated similar dynamics using multiphoton intravital imaging of glioma (*4*). In addition, our use of a maximum entropy approach comes with important caveats. While the model captures many statistical properties of the underlying experimental data, it should be viewed as an effective model, not necessarily a true physical representation of the underlying system. For example, the model suggests that pairwise interactions between cells are long-range, often extending across tens of cells or more, but there is no guarantee that the true biological interactions, whose mechanisms are, at this stage, largely unknown, extend over this scale. Instead, long-range interactions in the model represent effective interactions that appear artificially long range because (for example) the local cell dynamics have not reached equilibrium on the time scales of our measurements (*64*, *65*), meaning that the effective interactions represent many fast physical interactions between different pairs of cells. It is also worth stressing that the finding of criticality in the data-driven model is not, on its own, proof of criticality in the glioblastoma populations [it is well known that quantitative maximum entropy predictions do not necessarily reflect similar features in the underlying system (*39*, *66*–*71*)]. In any case, our primary claim, that fluctuations are scale-free, is based on direct the observation of correlations of the experimental system not on any particular model. We stress that these long-range correlations do not imply direct long-range physical cell-cell interactions.

In our glioblastoma models, growth is not constrained by the pattern of brain anatomy [i.e., white matter bundles and white matter/brain matter borders (*72*)], as growing rodent tumors replace brain tissue. Thus, in our model, the formation of oncostreams does not depend on the underlying brain structure (*73*); it is unclear whether they depend on the mechanics of surrounding tissue (*74*, *75*). The role of blood vessels in glioblastoma growth and response to treatment, as well as their potential role in oncostream dynamics, remains to be determined (*76*, *77*).

There are also a number of technical limitations that bear mention. We focused on a model with topological (rather than metric) connections between cells. For these populations, where the densities of cells are typically similar across populations, we expect that both approaches would yield similar results, although imposing a different coupling structure could improve the agreement between model and experiment (*10*). In addition, we have used free boundary conditions, partly because confining the boundary cells (using fixed boundary conditions) would, in some cases, reduce the number of cells for analysis. Previous work in starling flocks showed that these boundary conditions can make a difference in the inferred values of *J* and *n_c_*, although their product remains largely constant (*12*). Correcting for these boundary conditions might provide a more accurate fit to, for example, simulated systems with known control parameters. In this case, however, our goal was not to precisely estimate experimental parameters, and because the model captures experimental results reasonably well, we have not further investigated these issues.

Our results raise a number of open questions for future work. From a physics perspective, more detailed physical models may shed additional light on quantitative features of glioma migration that are not captured by the simple model used here. For example, we recently showed that a model of collective motion that incorporates cell shape can produce a rich collection of phenomena that includes nematic ordering and qualitatively distinct types of disordered motion (*7*) similar to that seen in glioma populations (*4*). Perhaps, our results imply the existence of (effective) long-range communication within glioma tumors, even if the physical basis for this communication is not yet understood. Previous work indicates that natural insect swarms exhibit long-range correlations (*21*), but these correlations seem to appear in response to different environmental perturbations in laboratory populations (*23*, *78*). Similarly, it may be possible to investigate these mechanisms in glioma populations using controlled laboratory conditions that strip away complexities of the ex vivo tumor environment. From a biological perspective, several mechanisms exist that could play a role in transmitting signals across large portions of glioma (*79*). For example, cytokines or neurotransmitters could be released by glioma cells, diffuse throughout the tumor tissue, and thus alter the behavior of distantly located cells. Equally, networks of microtubes connecting glioma cells have recently been described and shown to provide signals to other cells via calcium ion transients (*80*). So far, only shorter distance communication has been shown, but if microtubes are truly functional across larger distances, then they could be an anatomo-physiological substrate of long-range communication described herein. Furthermore, criticality could help understand and explore the recalcitrant robustness of these tumors when exposed to therapeutic modalities such as x-rays and chemotherapy. Criticality could play a role in glioma cells responding to resective surgery; for example, no matter how much tumor is resected, cells located within 1 to 2 cm from the resection cavity eventually reconstitute the tumor. Thus, it might be possible that low-density cells might need to grow to a certain population size before long-range communication can be exploited to support cell replication and especially cell invasion. Outcomes in the response to chemo- and radiotherapy could be tested experimentally by exposing brain slices to these treatments and then evaluating collective cell motility using ex vivo or in vivo imaging (*4*). Last, a role for the recently discovered brain innervation of tumors (*80*) could be to directly connect distant parts of the tumor, i.e., tumor cells could signal to innervating neurons that, through neural networks, would then signal back to distant tumor regions. We believe that the physical and mathematical modeling of glioblastomas has the potential to inspire further understandings of glioblastoma biology and potentially unique treatments aimed at destabilizing collective behavior.

## MATERIALS AND METHODS

### Time-lapse confocal imaging in explant brain glioma slice culture model

To analyze glioma cell dynamics, we used a brain tumor explant model for ex vivo imaging. In vivo experimentation was performed following the procedures approved by the Institutional Animal Care and Use Committee (IACUC) at the University of Michigan. Glioma tumor cells were obtained from a genetically engineered mouse glioma model generated in our laboratory as previously described (Sleeping Beauty Transposase System) (*45*–*47*). This model enables de novo generation of glioma tumors trough the injection of different plasmids encoding the genes of interest in postnatal day 1 (P01) wild-type C57BL/6 mice. The plasmid sequences used to generate these tumors were (i) the transposase and luciferase enzyme expression (pT2C-LucPGK-SB100X), (ii) the NRAS gene expression (pT2CAG -NRAS-G12V), (iii) the short hairpin for down-regulation of p53 protein (pT2-shp53-GFP4), and (iv) the short hairpin for ATRX (pT2-shATRx-GFP4). These glioma tumor cells named NPA exhibit the overexpression of the NRAS protein, the knockdown of p53, and the down-regulation of ATRX. shp53 and shATRx plasmids also contain the gene for the expression of enhanced GFP (EGFP). These signaling pathways are altered in human gliomas. Cells were generated from genetically engineered glioma tumors and cultured in Dulbecco’s minimum essential medium (DMEM)/F12 media supplemented with 2% B-27, 1% N-2, 1% of penicillin-streptomycin, 0.2% of Normocin, and growth factors human Fibroblast Growth Factor (hFGF) and human Epidermal Growth Factor (hEGF at 20 ng/ml and maintained in a 37°C incubator supplied with 95% air and 5% CO_2_. Tumors were induced by intracranial implantation of 3 × 10^4^ NPA cells (NRAGV12, shp53, and shATRx) glioma tumor cells in C57BL/6 mice. Mice were held in a pathogen-free, humidity- and temperature-controlled vivarium on a 12-hour light/12-hour dark cycles with free access to food and water following. Before implantation, mice were anesthetized using an intraperitoneal injection of the anesthetics ketamine (120 mg/kg) and dexmedetomidine (0.5 mg/kg). Following anesthesia, carprofen (5.0 mg/kg) was administered subcutaneously for pain management. The skull of the mouse was then immobilized in a stereotactic device. A burrhole was made using a 0.45-mm drill bit at coordinates corresponding to the striatum (0.5 mm anterior and 2 mm lateral from bregma). Cells were injected with a Hamilton syringe at a dorsoventral position of 3.5 mm ventral into the striatum. After injection, the incision was sutured and immediately following surgery, the animals were recovered from anesthesia using atipamezole via intraperitoneal injection (1.0 mg/kg) to reverse the effect of dexmedetomidine. A single subcutaneous injection of buprenorphine (0.01 mg/kg, subcutaneous) was administered as postoperative pain relief. Sutures were removed 10 days after surgery (*4*, *45*).

Tumors were allowed to grow for 19 to 21 days. At 19 to 21 days after implantation, animals were euthanized to generate the brain tumor slice explants for imaging. To generate brain tumor explants, brains were embedded in 4% low melting temperature agarose and kept on ice until solidification. Embedded brains were then immersed in ice-cold and oxygenated DMEM without phenol red and then sectioned in a Leica VT100S vibratome (Leica, Buffalo Grove, IL). Brain tumor sections (300 μm thick) were transferred to laminin-coated cell culture insert (Millipore Sigma, USA) placed into a 27-mm-diameter dish (Thermo Scientific) with DMEM/F12 media supplemented with 25% fetal bovine serum and penicillin-streptomycin. All steps were performed under sterile conditions in a BSL2 laminar flow hood. Tumor slices were then maintained in a cell culture incubator at 37°C with a 5% CO_2_ atmosphere. After 6 to 18 hours, medium was replaced with DMEM/F12 media supplemented with 2% B-27, 1% N_2_, 0.2% Normocin, penicillin-streptomycin (10.000 U/ml), and growth factors EGF and FGF (20 ng/ml). Tumor explants were then transferred to the incubator chamber of a single-photon laser scanning confocal microscope model LSM 880 (Carl Zeiss, Jena, Germany). For tumor imaging, the incubation chamber of the microscope was maintained at 37°C and 5% CO_2_. Images were acquired in a time-lapse frame of 10 min for 100 to 300 cycles. Raw data from movies used to perform the analysis described throughout this manuscript were originally described in (*4*).

### Image analysis

To track the evolution of the cells, we use the software Fiji with the plugin TrackMate. We use as parameters for the cell size (called “blob”) 20 μm and a threshold of 1 together with the DoG method (difference of Gaussian detectors). Each experiment gives several paths denoted by **x***_i_*(*t*) where *i* is an index for the cell and *t* represents the time. The paths are however erratic; thus, we apply a filter to smooth the trajectories over time (see Fig. 1). As a filter, we use a Gaussian kernel with SD σ^2^ = 2 and a stencil of 9 points

$${\bar{x}}_{i}(t)=\sum_{k=-4}^{4}\phi_{k}x_{i}(t-k\text{Δ}t),\phi_{k}=C\;\cdot e^{-k^{2}/4}$$ (1)

where ${\bar{x}}_{i}$ is the smooth trajectory, Δ*t* = 10 mn is the time step between successive image, and *C* is such that $\sum_{k=-4}^{4}\phi_{k}=1$. From the smooth trajectories ${\bar{x}}_{i}(t)$, we then estimate the velocities of the cells ${\bar{v}}_{i}(t)$ using a finite difference

$${\bar{v}}_{i}(t)=\frac{{\bar{x}}_{i}(t+\text{Δ}t)-{\bar{x}}_{i}(t-\text{Δ}t)}{2\text{Δ}t}$$ (2)

For each velocity vector **v***_i_*(*t*), we estimate a corresponding velocity angle θ*_i_*(*t*) ∈ [0,2π] (see Fig. 1). For subsequent analysis, all velocity vectors are normalized and referred to as ***s***_***i***_.

### Classification flock-stream-swarm

We subdivide glioma populations into connected subpopulations, which we refer to as flocks, streams, or swarms [see fig. S14 (left)]. This empirical classification scheme was developed in a previous work (*4*) to segment populations into subgroups with similar histological and statistical properties, as described briefly below. In the present context, these classifications are not particularly important, and instead, one may view this as a systematic way of subdividing populations for finite-size scaling, which is necessary because the number of experimental populations is limited [see (*52*) for further discussion of these issues in limited datasets].

To classify in which category an experiment is (i.e., flock, stream, or swarm), we analyze the collection of velocity angles {θ*_n_*}_*n* = 1. . *N*_ (where *N* is the sample size). We determine three densities for each pattern [see fig. S14 (right)]

$$\rho_{\mathrm{flock}}=\text{″}\mathrm{Gaussiandistribution}\text{″}$$ (3)

$$\rho_{\mathrm{stream}}=\text{″}\mathrm{symmetrizedGaussiandistribution}\text{″}$$ (4)

$$\rho_{\mathrm{swarm}}=\text{″}\mathrm{Constant}\text{″}$$ (5)

We then evaluate the likelihood of the velocity angles {θ*_n_*}_*n* = 1. . *N*_ given each distribution, for instance

$$L_{\mathrm{flock}}=\prod_{n=1}^{N}\rho_{\mathrm{flock}}(\theta_{n})$$ (6)

Last, we compare the three likelihoods (i.e., L_flock_, L_stream_, and L_swarm_) and select the pattern with the highest likelihood.

### Calculating correlation functions

The correlation function is given by *C*(*r*) = 〈δ*s_i_* · δ*s_j_*〉*_r_*, where δ*s_i_* ≡ *s_i_* − 〈*s*〉 is the velocity of cell *i* in the moving reference frame where the population average velocity (〈*s*〉) has been subtracted out. Angle brackets 〈〉*_r_* indicate an average taken over all cells separated by distance *r*. In practice, we calculated correlation functions by first calculating pairwise distances and dot products (δ*s_i_* · δ*s_j_*) for all pairs of cells in an image at a given time. We repeated this process for all images in a time series and combined the data from all time points into a single pair of lists (distances and corresponding dot products, ordered according to distance). The correlation functions are then calculated by smoothing using a moving average filter with a centered window of ±100 μm. Error bars/shading represent ±1 SEM within each window. Correlation functions (and the system-size scaling of correlations length) are qualitatively similar (although more or less noisy) for a range of window sizes from ±25 up to ±100 μm (fig. S3).

### Estimating system size (*L*)

To estimate the size *L* of each population, we calculated the maximum pairwise separation [*L*(*t*) ≡ *d*_max_] between any two cells at each time point. The distance *L*(*t*) will fluctuate over time as the cells move. We define the size of the system as the mean value of this separation distance over the entire time series [*L* ≡ 〈*L*(*t*)〉*_t_*]. We found qualitatively similar results (i.e., scaling of correlation length with system size) if we alternatively defined the system size at each time point as *L*(*t*) = *d*_con_, where *d*_con_ = *A*^1/2^ and *A* is the area of the convex hull of all cell positions S5.

### Box scaling analysis

To perform the box scaling analysis, we combined the data from all samples into a single dataset consisting of approximately 400 single-time snapshots of approximately 800 μm by 800 μm, each containing position and velocity information for hundreds of cells. For a given box size *W*, we selected all cells within a randomly positioned square box of length *W* from each snapshot. We then calculated the separation distances and dot products (δ*s_i_* · δ*s_j_*) for all pairs of cells within the box. The box sampling was repeated a total of 30 times for each snapshot, each with a newly positioned box, and the pairwise distance and dot product data were then combined to estimate a single correlation function *C_W_*(*r*) for systems in boxes of length *W*. We calculated these correlation functions for box sizes *W* ranging from approximately 200 to 800 μm and then used them to estimate a single characteristic length *r*_0_ for each box size, as described above in the section “Calculating correlation functions” (this time using a moving average filter with a centered window of ±50 μm).

### Nonlocally coupled *XY* model as a data-constrained maximum entropy model

To model collective motion in tumor cells, we used a maximum entropy framework. Maximum entropy models are required to match certain features of the data (in this case, the observed correlation *C*_int_, see below) but contain minimal additional statistical structure. Similar models have been previously used to describe firing patterns in neurons, immune system dynamics, interactions between antibiotics, and collective motion in starlings. Following (*12*), we constrain the model to match the scalar correlation over local neighborhoods of size *n_c_*, which is given by

$$C_{\mathrm{int}}=\frac{1}{N}\sum_{i=1}^{N}\frac{1}{n_{c}}\sum_{j\in n_{c}^{i}}{\bar{s}}_{i}\;\cdot {\bar{s}}_{j}$$ (7)

where *N* is the total number of cells, *n_c_* is the integer size of the local neighborhood, and ${\bar{s}}_{i}$ is the unit velocity vector describing the motion of cell *i*. The maximum entropy model consistent with this constraint is then given by (*12*)

$$P(\{{\bar{s}}_{i}\})=\frac{1}{Z(J,n_{c})}\exp\left(\frac{J}{2N}\sum_{i=1}^{N}\sum_{j\in n_{c}^{i}}{\bar{s}}_{i}\;\cdot {\bar{s}}_{j}\right)$$ (8)

where $P(\{{\bar{s}}_{i}\})$ is the distribution over all configurations and *Z*(*J*, *n_c_*) is the partition function (i.e., the normalization constant). To fully specify the model, we must choose values of the parameters *J* and *n_c_* such that the model reproduces the observed value of *C*_int_, a process that is equivalent to maximizing the likelihood that the model generates the configuration observed in a given snapshot of the flock.

To estimate model parameters, we first calculate the experimental correlation $C_{\mathrm{int}}^{\exp}$ (where the superscript “exp” indicates that this is the value observed in the experiment) for a single snapshot of the population. This experimental value must match the value of *C*_int_ produced by the model, which provides an explicit data-dependent constraint between the parameters *J* and *n_c_*

$$\frac{1}{J}=\frac{n_{c}}{2N}(1-C_{\mathrm{int}}^{\exp})$$ (9)

We then determine the remaining free parameter, *n_c_*, by numerically maximizing the log likelihood of the data given the model, which can be written as a function of the both $C_{\mathrm{int}}^{\exp}$ (a measured quantity) and *Z*(*J*, *n_c_*) (the partition function for the model).

Directly calculating the partition function *Z*(*J*, *n_c_*) is computationally expensive, even for this simple model. One option to simplify the calculation is to use a spin-wave approximation, which provides an analytical expression for *Z*(*J*, *n_c_*) in terms of eigenvalues of a matrix *A* that describes the neighborhood structure of the population (i.e., which cells are in the local neighborhood, of size *n_c_*, of each cell). In the spin-wave approximation, the partition function reduces to (*12*)

$$\log Z(J,n_{c})=-\sum_{k>1}\log\left(\frac{J\lambda_{k}}{N}\right)+\frac{Jn_{c}}{2}$$ (10)

where λ*_k_* are the eigenvalues of the matrix *A* = δ*_ij_*∑*_k_* ‍ *n_ik_* − *n_ij_*, where *n_ij_* is 1 if cell *i* is in the local neighborhood of cell *j* and vice versa, 1/2 if cell *i* is in the local neighborhood of cell *j* but *j* is not in the local neighborhood of cell *i* (or vice versa), and 0 otherwise. Note that the spin-wave approximation is strictly valid only in highly polarized populations, as it relies on neglecting higher-order terms in an expansion velocity components perpendicular to the direction of polarization. In this work, we used the spin-wave approximation to provide first-pass estimates of the parameters *J* and *n_c_*. While cell motion is not typically highly polarized in our dataset and the spin-wave approximation is therefore not strictly valid, we found that the parameters estimated from this approach do lead to strong agreement with the data.

### Estimating parameters from simulated data

To probe the reliability of parameters estimated with this spin-wave approximation, we used Monte Carlo simulations to produce artificial datasets, snapshots of velocity configurations for populations of cells whose positions are identical to those in the experimental data, drawn from the model with specified values of *J* and *n_c_*. We then estimated the parameters directly from the simulated data using either (i) the spin-wave approximation or (ii) a direct least squares fitting to observed distribution *P*({θ*_i_*}), where {θ*_i_*} is the configuration of velocity angles in the population.

### Mean field *XY* model

The maximum entropy model has two free parameters, *J* and *n_c_*, that are inferred from experimental data. In the limit *n_c_* → *N* − 1 ≈ *N* (all-to-all coupling), this model reduces to a mean field version of the 2D *XY* model with only a single free parameter, *J*. Estimating this parameter from data reduces to a classic inverse problem in statistical physics, with the log likelihood *L*(*J*) of the observed configurations taking the form

$$L(J)=\frac{1}{2}JNC_{\mathrm{int}}^{\exp}-\log Z(J)$$ (11)

where $C_{\mathrm{int}}^{\exp}$ is now the average pairwise correlation taken over the entire population. Maximizing the likelihood is equivalent to requiring that the correlations measured experimentally $\left(C_{\mathrm{int}}^{\exp}\right)$ match those from the mean field model, which reduces to a classical inverse problem. In this case, we can write down an explicit expression for *J* in terms of the experimental measurable

$$J=\frac{2}{\left(1-C_{\mathrm{int}}^{\exp}\right)}$$ (12)

### Estimating critical parameters in systems with long-range coupling

Equation 12 highlights an important caveat of our approach when the range of coupling *n_c_* is comparable to the total size of the system (*N*). The mean field *XY* model undergoes a phase transition at a critical value of *J_c_* = 2. Equation 12 therefore indicates that observed data characterized by $C_{\mathrm{int}}^{\exp}\approx 0$ would appear to be at a critical point of the mean field model. In practice, $C_{\mathrm{int}}^{\exp}\approx 0$ would also be expected for completely disordered populations, that is, for populations where the velocity direction is drawn from a uniform distribution. As a result, maximum likelihood estimates that indicate *J* ≈ *J_c_* are not, alone, sufficient evidence of criticality, as they cannot distinguish systems in the disordered region (*J* < *J_c_*) from those at criticality. To quantitatively characterize these limitations for datasets comparable in size to our experimental data, we used Monte Carlo simulations to generate artificial datasets representing cell populations globally coupled with different values of *J*. We then calculated the maximum likelihood estimates of *J* from those in silico datasets (just as we did with experimental data). As expected, we found that estimates of *J* hover around the critical value *J_c_* = 2 for simulated systems at or below the critical point (fig. S11). On the other hand, estimates of *J* are accurate for systems slightly above the critical point.

### Monte Carlo simulations and phase diagram

To characterize the phase diagram for the nonlocal *XY* model, we used Monte Carlo simulations to generate representative velocity distributions for different values of *n_c_* and *J*. For each parameter pair, we calculated the mean polarization across trials and the generalized susceptibility χ, which corresponds (up to a proportionality constant) to the variance of the polarization across trials. We then estimated the critical surface to be the curve in parameter space that corresponds to a peak in χ. We also verified that the system exhibits sharply increasing polarization and a peak in the generalized heat capacity at the critical surface. For all simulations, cell positions (and, therefore, cell density) were taken from a representative experimental dataset.

### Comparing velocity angle distributions between the model and data

To compare experimental results with results from the effective model, we calculated histograms of velocity angle. The model is symmetric under rotation and cannot provide information about the specific velocity direction, that is, we can globally rotate all velocities by an arbitrary angle. To compare histograms between the model and the data or, for example, to combine data from multiple independent simulations (model) or multiple time points (experiment), we first calculated the two frequency histograms to be compared. Then, we rotated all velocities in one population by an angle θ, and we tuned θ to achieve maximal alignment between the distributions (i.e., minimal difference in a least squares sense). Because the histograms themselves are noisy, this process could, in principle, lead to apparent similarities between distributions with fundamentally different shapes. Therefore, as a control, we simulated size-matched populations where velocity angles were drawn from a uniform distribution, and we then used this alignment process to compare these (nominally) uniform histograms with the histograms from the maximum entropy model. We confirmed that this alignment process does not yield substantial agreement between these different distributions (fig. S11).

## Notes

View/request a protocol for this paper from *Bio-protocol*.
